# Data from a randomized control trial that examined the impact of a cash transfer and psychological intervention on alcohol outcomes and household welfare in India

**DOI:** 10.1016/j.dib.2026.112914

**Published:** 2026-06-04

**Authors:** Joseph Ugbede Ejima, Parmod Kumar, Arjunan Subramanian

**Affiliations:** a Economics, Adam Smith Business School, University of Glasgow, Glasgow, UK; b Giri Institute of Development Studies, Lucknow, Uttar Pradesh, India; c Institute for Social and Economic Change, Bangalore, India

**Keywords:** Randomized controlled trial, Alcohol consumption, Blood alcohol content, Cash transfer

## Abstract

The present data article provides a dataset of daily alcohol consumption, an additional description of the participants, and measures of psychological hardiness and alcohol dependency related to the research article entitled “Cash Transfer and a Psychological Intervention: Impact on Alcohol Outcomes and Household Welfare in India” (Ejima et al., 2025). This randomized controlled trial examined the effect of a cash transfer and a psychological intervention on alcohol-related outcomes and household welfare. A total of 200 households with at least one frequent alcohol user were recruited and randomly assigned to one of four groups: a voucher intervention group that received a voucher for 30 days, a psychological intervention group that received five sessions of counselling, a joint intervention group that received both, and a control group. Data were collected during the baseline phase in December 2018 for all household members, including detailed demographic, income, and household consumption information. Within these households, 220 individuals were identified as frequent alcohol consumers and became the main study participants. For these participants, data on daily alcohol consumption, blood alcohol content (BAC), and alcohol-related expenditure were collected over a 90-day period. Additionally, psychological hardiness and alcohol dependency, measured using the validated CAGE questionnaire, were assessed at both baseline and endline.

Specifications Table

**Table:** 

Subject	Social Sciences
Specific subject area	Health and Experimental Economics
Type of data	Table Processed data Data format (.csv)
Data collection	Quantitative data were collected through a structured baseline household survey conducted in December 2018. This survey gathered detailed information on demographics, income, assets, and consumption from all household members, along with individual-level data on alcohol and smoking behaviors. Based on the baseline findings, 220 individuals identified as frequent alcohol consumers were enrolled in a 90-day follow-up daily monitoring study conducted from January 5 to April 4, 2019. During this period, participants recorded their daily alcohol consumption, expenditure, and blood alcohol concentration (BAC) using handheld breath analysers. Additionally, these individuals completed the CAGE questionnaire (to assess potential alcohol dependency) and a psychological hardiness scale at both baseline (December 2018) and endline (April 2019). All surveys were administered through in-person interviews, enabling direct interaction between researchers and participants and ensuring consistency and reliability in data collection.
Data source location	The data were collected in three randomly selected villages in Patiala, Punjab, India: Ajnaudha Kalan, Dhanauri, and Simbro.
Data accessibility	Repository name: Mendeley Data Data identification number: https://dx.doi.org/10.17632/bpyzmjrcv8.3
Related research article	J. U. Ejima, P. Kumar, A. Subramanian, Cash transfer and a psychological intervention: impact on alcohol outcomes and household welfare in India, Soc. Sci. Med. 382 (2025) 118358. https://doi.org/10.1016/j.socscimed.2025.118358

## 1 Value of the Data

• This dataset contains detailed, high-frequency information on alcohol-related outcomes collected over a 90-day period, enabling analysis of short-run consumption patterns and immediate behavioural responses to an intervention.
• The inclusion of daily blood alcohol content (BAC) data provides an objective proxy for alcohol exposure, reducing reliance on self-reported measures and potential reporting bias.
• The high temporal resolution allows researchers to examine within-person variation and short-term dynamics in alcohol use. Intended users include: (i) researchers studying short-run responses to interventions targeting alcohol use; (ii) researchers interested in the validation of objective versus self-reported alcohol measures in low-resource settings; (iii) economists and public health scholars examining within-person consumption dynamics and high-frequency behavioral variation; and (iv) researchers developing econometric or statistical methods for analyzing noisy high-frequency outcomes. The dataset is less suited for evaluating long-run behavioral change, alcohol-related harms, or clinically defined heavy episodic drinking due to the 90-day observation horizon and outcome coverage.

## 2 Data Description

This dataset contains detailed longitudinal data on alcohol consumption and expenditure for 220 individuals identified as frequent alcohol consumers and enrolled in a randomized controlled trial to assess the impact of cash voucher and psychological counselling interventions on alcohol use and related behaviours. Study households were recruited through direct, in-person visits conducted by trained field enumerators, who assessed household eligibility during structured visits within each study village. Fieldwork was carried out by multiple teams consisting of experienced researchers and locally recruited assistants from the community, under the technical supervision of academic staff from the Department of Psychology at Patiala University, who ensured adherence to the study protocol and data collection procedures. All participants provided informed consent prior to enrolment through a standardized consent process that clearly explained the objectives, procedures, and duration of the study, and no direct financial incentives were offered for participation. It should be noted that two members of the author team had conducted previous research in these villages, and that 32% of households in the current sample had participated in an earlier survey, which may give rise to response or familiarity effects. However, these data were generated within a randomized controlled trial with random assignment across treatment arms, implying that any such effects are expected to be balanced by design in the underlying data-generating process and therefore unlikely to systematically distort the overall structure of the dataset. Nevertheless, these factors may still have implications for baseline responses or external validity, which users should consider when utilizing the data.

At both baseline and endline, data were collected on demographic characteristics (age, sex, relationship to household head), education level, main and secondary occupation, caste, religion, village identifiers, and economic variables (see Table 1). The economic variables include household asset ownership and landholding. Tobacco use was also measured through self-reported smoking status, type of product consumed, and number of packs (see Table 1). In addition, high-frequency daily alcohol consumption data were collected from each participant over a 90-day period. These daily records include the day and date of observation, total quantity of alcohol consumed (in millilitres), daily alcohol expenditure, time of last drink, time of BAC measurement, and blood alcohol content recorded using a handheld breathalyser. To facilitate comparability with studies reporting intake in grams of ethanol or standard drink units, volumes expressed in millilitres can be converted using WHO conversion factors (see [1], p. 45). Psychological and behavioural measures were collected using two instruments. Alcohol dependence was assessed using the CAGE questionnaire, a widely used and validated screening tool comprising four binary items. Evidence from validation studies indicates that the CAGE performs well in detecting alcohol abuse and dependence in clinical populations, although its performance in general and primary care populations is more variable [2]; we therefore interpret CAGE scores conservatively as a screening indicator rather than as a diagnostic measure in this community-based sample. Psychological hardiness was measured using a 12-item author-constructed scale, adapted from the psychological hardiness framework introduced by Kobasa [3] and subsequent work by Maddi [4]. This measure is not a previously validated instrument but was developed for the purposes of this randomized controlled trial to capture relative variation in hardiness-related attitudes within the study population. Psychological hardiness is conceptualized as a personality trait comprising three core components - commitment, control, and challenge - which reflect individuals’ tendencies to remain engaged, perceive control over outcomes, and view difficulties as opportunities for growth [4].

**Table 1:** Additional characteristics of participants modified from [5].

**Panel A: Continuous variables**	Obs	Mean
Age	220	45.777
Total household assets (₹)	220	98148.640
Total acres of land	220	5.606
Number of packs smoked	135	25.570

**Panel B: Categorical variables**	Frequency	Percent

**Gender**		
Male	216	98.18
Female	4	1.82
**Household head**		
Yes	160	72.73
No	60	27.27
**Level of education**		
Illiterate	70	31.82
Below primary	9	4.09
Primary	32	14.55
Middle	41	18.64
Secondary/Metric	44	20.00
Technical	22	10.00
Graduate	2	0.91
**Main occupation**		
Self-employed farming	44	20.00
Self-employed non-farming	19	8.64
Salary	33	15.00
Pensioner	2	0.91
Agricultural wages	49	22.27
Non-agricultural wages	56	25.45
Dependent	13	5.91
Household work	4	1.82
**Village**		
Ajnaudha Kalan	74	33.64
Dhanauri	69	31.36
Simbro	77	35.00
**Caste**		
General caste	78	35.45
Other backward caste	53	24.09
Scheduled caste	85	38.64
Others	4	1.82
**Religion**		
Hindu	29	13.18
Muslim	10	4.55
Ravidas	1	0.45
Sikh	180	81.82
**Currently smoking status**		
Yes	134	60.91
No	86	39.09
**Type of product smoked**		
Bidi	69	51.11
Cigarette	5	3.70
Tobacco	61	45.19

We provide four CSV data files. The first file, “Demographics.csv”, includes all baseline information on participant characteristics as described in Table 1, including details on smoking habits. The second file, “Daily Alcohol Measures.csv”, contains high-frequency daily data collected over a 90-day period for each participant. The third file, “Psychological Hardiness Survey.csv”, provides data from a 12-item psychological hardiness scale for each participant, available at both baseline and endline, which can be used to construct a hardiness index. The fourth file, “CAGE Survey.csv”, contains both baseline and endline responses to the CAGE questionnaire, which can be used to determine alcohol dependence among participants. The construction of the hardiness index and the measurement of alcohol dependence are described in the supplementary materials of Ejima et al. [5], the study from which this data paper is derived.

All four datasets can be merged using the unique identifier assigned to each respondent. In addition, household identifiers in the demographic data allow researchers to identify individuals belonging to the same household and to account for within-household clustering. Detailed variable definitions are provided in the accompanying README file.

## 3 Experimental Design, Materials and Methods

This was a randomized trial conducted in three villages in Patiala, Punjab, to assess the effects of financial and psychological interventions on alcohol-related outcomes among frequent alcohol consumers. Eligible households were identified through a baseline survey in December 2018 that collected demographic, income, consumption, and individual alcohol use data. All survey data were collected through face-to-face interviews administered by trained field enumerators using structured questionnaires, and no instruments were self-administered by participants. Households with at least one frequent alcohol consumer were invited to participate, and written informed consent was obtained. A total of 200 households were randomly assigned at the household level to one of four groups: control (n = 65), voucher intervention (n = 45), psychological counselling intervention (n = 45), and combined voucher and counselling intervention (n = 45).

In the voucher intervention group, participants received daily vouchers worth ₹50 (in ₹20, ₹20, ₹10 denominations) for 30 days, redeemable at local shops and contracted liquor stores. Voucher usage was tracked directly by field staff. In each village, one designated shop was assigned to accept vouchers as payment, and contracted liquor stores located outside the villages also accepted vouchers. Shopkeepers submitted collected vouchers for reimbursement on a daily basis, and during these reimbursement visits enumerators recorded detailed information on voucher use, including the type of commodity purchased and the value redeemed, which was documented in a voucher utilization diary maintained by the research team.

In the psychological counselling intervention group, five structured sessions were delivered at regular intervals by trained psychologists. The sessions incorporated Prochaska’s Stages of Change model, Acceptance and Commitment Therapy (ACT), and the RESISTT impulse-control technique [6]. Sessions focused on building motivation to reduce alcohol use, teaching coping strategies, and reinforcing behaviour change. The joint intervention group received both the voucher and psychological counselling interventions according to the same protocol as the individual treatment groups. The control group received no intervention but participated in all baseline and follow-up data collection activities.

## Limitations

One important limitation of the dataset pertains to the timing of blood alcohol content (BAC) measurements. On days when logistical constraints prevented field staff from administering the breathalyser test within the same calendar day as alcohol consumption, measurements were deferred to the following morning. This introduces potential bias, as BAC levels decline over time due to metabolic clearance, thereby compromising the precision of alcohol exposure assessment for the prior day. As a result, BAC values recorded under these conditions may underestimate actual intoxication levels. Using clock-time information, we identify that approximately 0.82% of BAC measurements were conducted after midnight relative to the reported time of last drink. In addition, while self-reported alcohol consumption measures may be subject to response bias, the inclusion of objective BAC measurements helps to mitigate reporting-related bias. Nevertheless, repeated objective measurement may induce behavioural adaptation, whereby participants alter their consumption in response to being monitored. While we cannot rule this out, the BAC outcome should be interpreted as a high-frequency proxy for short-run alcohol exposure, rather than a precise measure of unconstrained daily behaviour.

## Ethics Statement

Ethical approval was given by the Institutional Ethics Committee of ISEC, Bangalore, with reference number DPA/Ethics Com./2018/542. Informed consent was obtained from all participants prior to data collection, following a clear explanation of the study’s purpose, procedures and participants’ right to withdraw at any time without penalty.

## CRediT authorship contribution statement

**Joseph Ugbede Ejima:** Conceptualization, Data curation, Formal analysis, Investigation, Methodology, Software, Writing – original draft, Writing – review & editing. **Parmod Kumar:** Conceptualization, Data curation, Investigation, Project administration, Writing – review & editing. **Arjunan Subramanian:** Conceptualization, Data curation, Funding acquisition, Investigation, Methodology, Supervision, Writing – review & editing.

## Data Availability

Mendeley DataData From a Randomized Control Trial That Examined the Impact of a Cash Transfer and Psychological Intervention on Alcohol Outcomes and Household Welfare in India, 2018-2019 (Original data)
